# Female rats have a different healing phenotype than males after anterior cruciate ligament rupture with no intervention

**DOI:** 10.3389/fmed.2022.976980

**Published:** 2022-11-14

**Authors:** Jodie L. Morris, Hayley L. Letson, Erik Biros, Peter C. McEwen, Geoffrey P. Dobson

**Affiliations:** ^1^Heart and Trauma Research Laboratory, College of Medicine and Dentistry, James Cook University, Townsville, QLD, Australia; ^2^Orthopaedic Research Institute of Queensland, Townsville, QLD, Australia

**Keywords:** anterior cruciate ligament, ACL rupture, sex differences, inflammation, healing, tissue remodeling

## Abstract

Little is known on the sex-specific healing responses after an anterior cruciate ligament (ACL) rupture. To address this, we compared male and female Sprague-Dawley rats following non-surgical ACL rupture. Hematology, inflammation, joint swelling, range of motion, and pain-sensitivity were analyzed at various times over 31-days. Healing was assessed by histopathology and gene expression changes in the ACL remnant and adjacent joint tissues. In the first few days, males and females showed similar functional responses after rupture, despite contrasting hematology and systemic inflammatory profiles. Sex-specific differences were found in inflammatory, immune and angiogenic potential in the synovial fluid. Histopathology and increased collagen and fibronectin gene expression revealed significant tissue remodeling in both sexes. In the ACL remnant, however, Acta2 gene expression (α-SMA production) was 4-fold higher in males, with no change in females, indicating increased fibroblast-to-myofibroblast transition with higher contractile elements (stiffness) in males. Females had 80% lower Pparg expression, which further suggests reduced cellular differentiation potential in females than males. Sex differences were also apparent in the infrapatellar fat pad and articular cartilage. We conclude females and males showed different patterns of healing post-ACL rupture over 31-days, which may impact timing of reconstruction surgery, and possibly clinical outcome.

## Introduction

Anterior cruciate ligament (ACL) rupture is one of the most debilitating musculoskeletal injuries and typically occurs following excessive rotational stresses and/or extreme deceleration forces (1, 2). Globally, there are over 2 million ACL injuries each year, with annual incidence rates continuing to rise, particularly in the young (2–5). Although females are 2 to 8 times at higher risk for ACL injuries than men (6–8), it is not known if there are sex differences during early healing following a rupture (2, 6–9). Some studies indicate that females may be more susceptible to functional deficits after ACL reconstruction (ACLR) surgery than men (10, 11), however, few studies have focused on the impact of sex on early healing. This is an important clinical question because there is much controversy about how long one should wait before ACLR surgery (9, 12). Too early can increase the risk of post-operative arthrofibrosis, and too late can increase the risk of meniscus tear and cartilage injury (1). Timing appears to be patient-specific and depends upon location and extent of the rupture, size of initial gap and proximity to bone, joint swelling, pain, range of movement, strength and leg control, as well as the patient’s psychological preparedness (12). The aim of our study was to characterize the systemic inflammatory responses and local healing processes within the ACL remnant, and adjacent joint tissues, in males and females over a 31-day period in a rat model of non-surgical ACL rupture. Based on studies after major trauma, (6, 13–15), we hypothesized that sex differences would exist in the healing environment within the ACL-ruptured knee in the absence of any intervention. This is the first of a larger body of continuing work evaluating joint tissue healing after ACL rupture, the effect of ACLR surgery, and the implications of sex and timing of surgical intervention on postoperative outcomes.

## Materials and methods

### Study design

**FIGURE 1:**
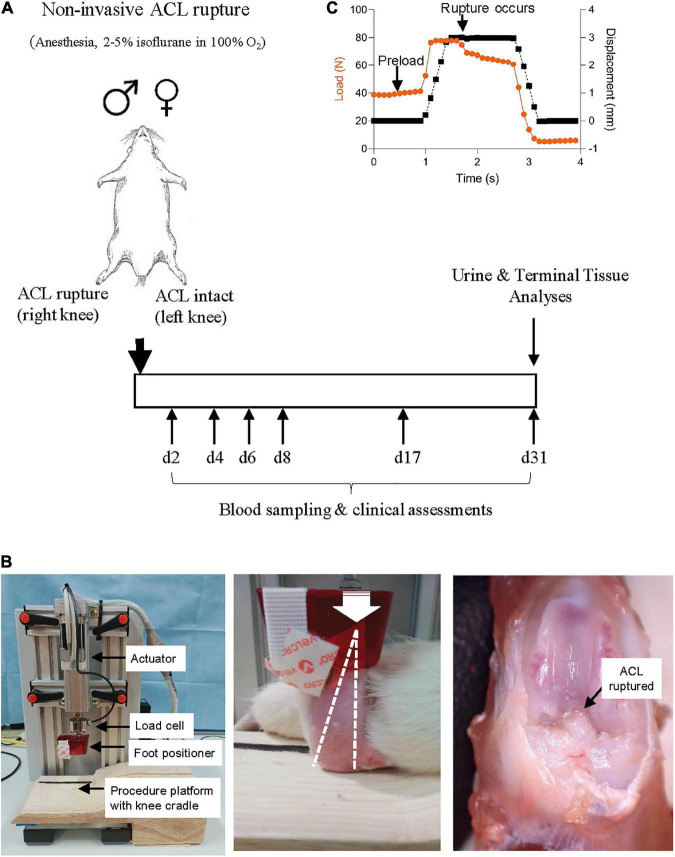
**(A)** Study protocol schematic. Non-surgical ACL rupture was performed on the right knee of anesthetized male and female Sprague Dawley rats. Left knees underwent sham injury, with the ACL remaining intact. At day 31 post-ACL rupture, animals were euthanized for terminal tissue analyses. **(B)** Custom non-surgical rat ACL rupture apparatus (left panel). The anesthetized animal was placed in a prone position on the procedure platform, the right hind limb secured with the knee in 100° flexion (white dotted line, middle panel) and the ankle in 30° dorsiflexion within the foot positioner. A compressive load was applied through the knee (white arrow indicates direction of load) at 8 mm/s with a displacement of 2.5–3 mm, resulting in ACL rupture (black arrow, right panel). **(C)** ACL rupture is evident by a quasi-instantaneous drop in load on the data stream.

Conventional, 16-week old male (*n* = 10) and female (*n* = 10) Sprague-Dawley rats were purchased from the James Cook University Small Animal Facility for these studies. Non-surgical ACL rupture was performed with post-injury assessment over a 31-day experimental period (Figure 1A). Animals were housed in ventilated cages (Tecniplast^®^ Australia, NSW, Australia) in a 14–10 h light-dark cycle under controlled temperature (21–22°C) and humidity (65–75%) conditions, with access to standard rodent pellets (Specialty Feeds, WA, Australia) and water *ad libitum*. Animals were acclimated for at least 7 days prior to experimentation. All animal experiments followed protocols approved by the James Cook University Animal Ethics Committee (A2684) and the US Animal Care and Review Use Office (ACURO), and were conducted and reported according to the Animal Research: Reporting of *in vivo* Experiments (ARRIVE) guidelines.

Animals were randomized to whether end-point tissue samples were to be fixed (histology, *n* = 5 per group) or snap-frozen in liquid nitrogen (gene and protein expression, *n* = 5 per group). Clinical signs including body weight, temperature and weight-bearing activity were monitored daily throughout the experimental period. Clinical and joint function assessments, and blood sampling was performed by a single researcher to avoid inter-use variation. Animals were euthanized 31 days post-ACL rupture with an overdose of pentobarbital (100 mg/kg) for gross pathology, molecular and histological evaluation.

### Non-surgical anterior cruciate ligament rupture

A custom non-surgical ACL rupture apparatus was constructed (CMA Electro-Hydraulic Engineers, Illawara, NSW, Australia), comprising an electric linear actuator and controller (LEY16C-50-R16P1, SMC World, Sydney, NSW, Australia) with load cell (HBM C9C, NVMS, Paramatta, NSW, Australia), an aluminum test frame, a custom acrylic foot cradle and procedure platform (Figure 1B). LabJack software (2019-05-20, LabJack Corporation, Lakewood, CO, USA) was used to configure and stream data from the datalogger, and ACT controller (SMC World) software was used as the control interface for the actuator.

On the day of ACL rupture, animals were anesthetized with isoflurane 5% (in 100% oxygen) during the induction phase and maintained at 1.5–2.5% until recovery, with animals breathing spontaneously. Animals were placed in a prone position, the right knee positioned and stabilized in 100° flexion and the right hind paw positioned in 30° dorsiflexion in a custom cradle centered under the linear actuator apparatus (Figure 1B). A pre-load was applied through the foot position for 5 s to stabilize the hind limb on the procedure platform at the desired angles, prior to ACL rupture. Anterior tibial subluxation was performed by applying a single compressive load to the flexed knee joint at a loading rate of 8 mm/s to achieve 2.5–3 mm vertical displacement, with endpoint defined by an audible popping sound upon ligament rupture and visualization of a quasi-instantaneous drop in load on the data stream (Figure 1C). ACL rupture was confirmed by anterior drawer test and by gross morphological examination at the end of the experimental period. The contralateral limb (left) of each animal was secured into the foot cradle and knee cup and a load of 10–15N was applied to the flexed limb for 5 s, however no compressive load was applied (ACL intact) (sham injury). Analgesia [Carprieve^®^ (Carprofen), 5 mg/kg, s.c. in 1 ml saline] was administered within 15 min of ACL rupture, and immediately prior to recovery from anesthesia. ACL rupture parameters [maximum load, maximum displacement and loading curve slope (max load minus preload/displacement)] were calculated for each animal. Following recovery from anesthesia, animals were free to weight-bear when ready to do so.

### Hematology and inflammatory assessments

Blood was collected under anesthesia via the lateral tail vein during the experimental period or via terminal cardiac puncture at day 31 post-injury (*n* = 10 per group) (Figure 1A). Post-ACL rupture metrics were compared to baseline hematology and inflammatory cytokine parameters from age-matched, healthy animals (*n* = 10 male, *n* = 10 female) that had not undergone anesthesia or injury. Complete blood cell examination (CBE) was carried out using a VetScan HM5 hematology analyzer (Abaxis, CA, USA). Blood samples were centrifuged and plasma collected and stored at -80°C until further analysis. Inflammatory chemokines and cytokines were measured in plasma [interleukin (IL)-1α, IL-1β, IL-2, IL-4, IL-6, IL-10, IL-12p70, IL-13, tumor necrosis factor (TNF)-α, interferon (IFN)-γ, monocyte chemoattractant protein (MCP)-1, macrophage inflammatory protein (MIP)-1α, regulated upon activation, normal T cell expressed and presumably secreted (RANTES)] and synovial wash [analytes measured in plasma plus granulocyte colony-stimulating factor (G-CSF), granulocyte-macrophage colony-stimulating factor (GM-CSF), growth regulated protein/keratinocyte chemoattractant (GRO/KC), lipopolysaccharide-inducible CXC chemokine (LIX), fractalkine, IL-17A, IL-18, interferon-inducible protein (IP)-10, and vascular endothelial growth factor (VEGF)] using custom Milliplex^®^ Rat Cytokine/Chemokine Magnetic Bead Panels (Abacus ALS, Cannon Hill, QLD, Australia), as described previously (16).

### Joint swelling and static joint angle measurement

Joint swelling was assessed at day 2, 4, 6, 8, 17, and 31 post-injury (*n* = 10 per group) by measuring the diameter (medial-lateral) of ACL-ruptured (right) and ACL-intact (left) knees with digital calipers in triplicate (16). Data show the difference in size between the injured and non-injured knee following ACL rupture. The knee extension angle, or the angle between the longitudinal axis of the femur and the tibia, was measured in triplicate using an angulometer with 20 g force for both the ACL-ruptured (right) and ACL-intact (left) knee at day 2, 8, 17, and 31 post-injury (*n* = 10 per group) (16). Data show the difference in extension angle between the injured and non-injured knee following ACL rupture.

### Sensory testing

The paw withdrawal threshold was assessed in the ACL-ruptured (right) and ACL-intact (left) knee (*n* = 10 per group) at days 2, 8, 17, and 31 using an up-down protocol for von Frey filaments (17). After a 5-min acclimation period on a wire-bottom cage, a graded series of 5 von Frey filaments (1.4–15 g of force) was applied in ascending order to the mid-plantar surface of the hind paws. Each filament was applied to the point of bending four times each on the hind footpad of the injured and control leg. The smallest filament eliciting a paw withdrawal response was considered the threshold stimulus. Data show the difference in threshold stimulus between the injured and non-injured limb following ACL rupture.

### Tissue collection

Following blood collection at day 31 post-injury, animals were euthanized to assess joint tissue metrics. Macroscopic morphological analysis of the intact joint, the articular surfaces, and capsular and synovial tissue was performed using methods described previously (16). One subgroup of animals (*n* = 5 male, *n* = 5 female) was perfused with ice-cold 1M phosphate-buffered saline (PBS, pH 7.4) followed by 4% paraformaldehyde (PFA). Left and right hind limbs with knee joints intact were fixed in 4% PFA for 48 h then used for histological assessment (see below). In parallel animals (*n* = 5 male, *n* = 5 female), the ACL-ruptured (right) and ACL-intact (left) knee of each rat was exposed, keeping the articular capsule intact. Lavage of the synovial cavity of the knee joint was performed with a total of 0.06 ml of physiological saline. The douche fluid was collected, snap frozen in liquid nitrogen and stored at −80°C for subsequent analysis of inflammatory cytokines and chemokines. Knees were then dissected to assess for macroscopic damage to major joint structures and the mode of ACL rupture. ACL rupture was classified as a mid-substance rupture, or avulsion at the tibial or femoral insertion. Tissue samples of the ACL remnant, infrapatellar fat pad (IFP), and cartilage of the medial femoral condyle (MFC, anterior and posterior aspects) and medial meniscus (MM) were collected, snap-frozen and stored at −80°C for subsequent RNA/protein extraction and analysis.

### RNA isolation and quantitative RT-PCR

Total RNA was isolated from joint tissue samples and cDNA was prepared by reverse transcription, as described previously (16). Real-time PCR with custom-designed primers was used to assess gene expression of key markers of inflammation [nuclear factor kappa B subunit 1 (Nfkb1), arginase 1 (Arg1)]; extracellular matrix (ECM) constituents [collagen type 1 alpha 1 chain (Col1a1), collagen type III alpha 1 chain (Col3a1), fibronectin 1 (Fn1), elastin (Eln), aggrecan (Acan), cellular communication network factor 2 (Ccn2)]; ECM remodeling enzymes [matrix metallopeptidase 9 (Mmp9), ADAM metallopeptidase with thrombospondin type 1 motif (Adamts4), tissue inhibitor of matrix metalloproteinase 1 (Timp1)]; and myofibroblast activation and differentiation [transforming growth factor beta 1 (Tgfb1), actin alpha 2 (Acta2), peroxisome proliferator-activated receptor gamma (Pparg)] (Supplementary Table 1). The relative expression of each gene was calculated using the concentration-Ct-standard curve method and normalized using the average expression of the housekeeping hypoxanthine-guanine phosphoribosyl tranferase (Hprt1) gene for each sample (16).

### Histology

For routine histology, fixed intact knees were decalcified (14% EDTA), processed and paraffin-embedded. Sections (4 μm) were cut in the frontal plane, spaced at 50 μm intervals and spanning the entire knee joint (18). Stained sections were visualized with light microscopy (Nikon Eclipse i50; Japan) and digitized for analysis using ImageJ^®^ software (v1.52p; National Institutes of Health, EUA). Semi-quantitative evaluations were performed by a blinded investigator on 4 non-adjacent sections for each knee. Synovitis in the IFP and cellularity within the ACL remnant were assessed on hematoxylin and eosin (H&E)-stained sections, using a previously described grading system (16). Masson-Goldner trichrome staining was used to visualize content and organization of collagen within the ACL remnant. Immunohistochemical staining of α-SMA in ACL remnants was performed using methods described previously (16). Cartilage degradation was assessed in the MFC, MM and medial tibial plateau using toluidine blue staining and scored using the Osteoarthritis Research Society International (OARSI) recommendations for histologic assessment of osteoarthritis in the rat (18).

### Statistics

*A priori* power analysis was conducted using G*power 3 program (Heinrich Heine University Düsseldorf) to determine sample size with effect size for outcome measure Nfkb expression in rat joint tissue in a pilot study (Allocation ratio N2/N1 = 1; Cohen’s *d* = 2.28; α err prob = 0.05; sample size *n* = 5; Power (1-β err prob) = 0.88). Statistical analyses were performed using GraphPad Prism software (version 9.0.0). Data normality was assessed using Shapiro-Wilks test, with Levene’s test used to determine equality of variances. Two-way ANOVA with Sidak’s multiple comparisons test were used for between groups comparison for normally distributed data (hematology parameters, inflammatory marker concentrations). Non-normally distributed data (macroscopic and histology scores, clinical assessments of joints) were compared using a Mann-Whitney *U*-test. MILLIPLEX Analyst 5.1 software (Luminex Corporation, Austin, Texas, USA) was used to determine cytokine and chemokine concentrations with a 5-parametric logistic weighted curve fit. Results are expressed as mean ± standard error (SEM) unless otherwise stated, with significance set at *p* < 0.05.

## Results

### Anterior cruciate ligament rupture profiles and post-injury recovery

Non-surgical ACL rupture was performed on the right knee of anesthetized male and female rats (Figure 1). No adverse events occurred and no sex differences were observed for the maximum load, maximum displacement, or loading slope (Table 1). The distribution of ACL failure modes was similar for both sexes, involving proximal (male, *n* = 9; female, *n* = 8) or mid-substance (male, *n* = 1; female, *n* = 2) tears. No meniscal or ligamentous defects were noted upon gross dissection at day 31 following ACL rupture.

**TABLE 1:** ACL rupture parameters for male (*n* = 10) and female (*n* = 10) rats.

Parameter	Male	Female	*P-value*
Maximum load (N)	77.9 (0.04)	74.9 (7.3)	0.120
Maximum displacement (mm)	2.7 (15.2)	2.8 (14.3)	0.712
Loading slope (N/mm)	16.8 (18.6)	19.3 (19.1)	0.119

Data show mean (coefficient of variation, %). Student’s *t*-test with Welch’s correction.

Minor weight loss was observed following ACL rupture, however, all animals returned to, and exceeded pre-injury weights within 21 days of injury (Supplementary Figure 1). There were no signs of lameness for any animal following ACL rupture, with all animals weight-bearing immediately following injury.

### Sex differences in hematology responses

**FIGURE 2:**
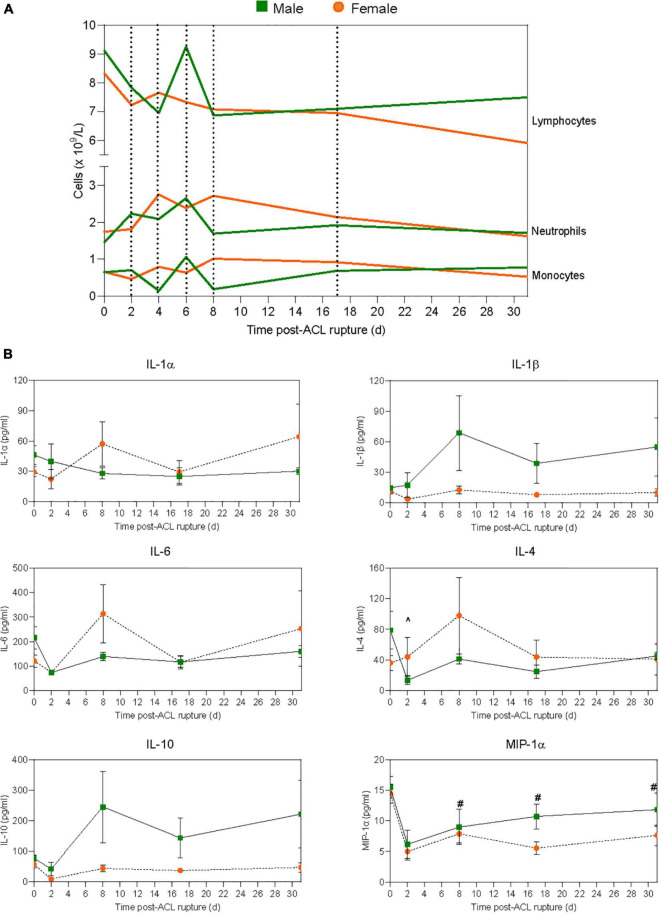
Systemic inflammatory responses to ACL rupture. Changes in **(A)** circulating lymphocyte, neutrophil, and monocyte numbers, and **(B)** plasma cytokine levels for male (green) and female (orange) animals to day 31 following ACL rupture. IL, interleukin; MIP, macrophage inflammatory protein. Data show mean (hematology) and mean ± SEM (cytokines). Two-way ANOVA, Sidak’s multiple comparisons test, ^∧^*p* < 0.05 male, compared to baseline. ^#^*p* < 0.05 female, compared to baseline.

Changes in complete blood cell counts following ACL rupture are shown in Figure 2A and Supplementary Table 2. Total leukocyte numbers remained comparable to baseline for both males and females across the experimental period, with no significant sex differences (Supplementary Table 2). At 2 days after ACL rupture, minor decreases occurred in circulating lymphocytes and monocytes in both sexes (ns; Supplementary Table 2). In contrast, neutrophils (154% increase; *p* > 0.05) and neutrophil-to-lymphocyte ratio (NLR; 75% increase; *p* = 0.033) were slightly elevated above baseline at day 2 for males, though differences between sexes were not statistically significant (Figure 2A and Supplementary Table 2). By day 4 post-injury, while monocyte numbers returned to baseline levels in females, further decreases (∼85%) occurred in males, however sex differences were not statistically significant. Circulating lymphocyte and monocyte counts returned to baseline levels for male and females by day 6, with minor elevations in neutrophils persisting for both (males, ∼75%; females, ∼41%; *p* > 0.05; Figure 2A). However, at day 8 the proportion of monocytes was significantly lower in males (*p* = 0.009) than females, despite similar levels of lymphocytes. In contrast, neutrophils and NLR tended to be higher in females than males at day 8, though these changes were not statistically significant (Figure 2A and Supplementary Table 2). By day 17, circulating monocyte and neutrophil numbers were similar for males and females, with levels returning to, and remaining at baseline levels to day 31 post-injury for both (Figure 2A and Supplementary Table 2). Lymphocyte numbers tended to remain below baseline for males and females between day 17 (∼22% vs. ∼17%, respectively) and 31 (∼17% vs. ∼29%, respectively) post-injury, though differences were not statistically significant (Figure 2A and Supplementary Table 2).

Following ACL rupture, platelet numbers remained comparable to baseline levels for both males and females across the experimental period, although at day 8 platelets were significantly higher in males than females (*p* = 0.027; Supplementary Table 2). Compared to baseline, red blood cell numbers were significantly increased in males following ACL rupture, and significantly higher than females at days 6–31 (Supplementary Table 2). Hematocrit decreased significantly for both sexes, and was significantly lower in females than males at day 6 (*p* = 0.002) and 8 (*p* < 0.001) post-injury. Similarly, hemoglobin was lower in females than males at day 6 (*p* = 0.005) and day 8 (*p* = 0.002) following ACL rupture, with levels returning to baseline by day 17 (Supplementary Table 2).

### Sex differences in plasma cytokine responses

At day 2 after ACL rupture, IL-1β decreased 3-fold in females, then returned to baseline levels by day 8 and remained stable over the remainder of the experimental period (Figure 2B and Supplementary Table 3). In contrast, IL-1β increased in males following ACL rupture and although not statistically significant, tended to remain higher than females from day 8 to 31. IL-1α, however, remained below baseline levels for males after ACL rupture, while minor increases were observed in IL-1α in females at day 8 (1.9-fold) and day 31 (2.2-fold) (*p* > 0.05; Figure 2B and Supplementary Table 3). Similar subtle changes were observed for IL-6, with minor increases above baseline concentrations at day 8 (2.6-fold) and day 31 (2.1-fold) post-injury in females only (*p* > 0.05; Figure 2B and Supplementary Table 3). Plasma IL-4 decreased significantly from baseline at day 2 post-injury in males (*p* = 0.006), and remained 1.7-fold lower than baseline at day 31. In contrast, IL-4 levels remained similar to baseline values in females, with a 2.4-fold increase at day 8 (*p* > 0.05; Figure 2B and Supplementary Table 3). For IL-10, the opposite occurred, with levels below baseline throughout the experimental period in females, and above baseline in males at day 8 (3.1-fold), day 17 (1.8-fold), and day 31 (2.8-fol) (*p* > 0.05; Figure 2B and Supplementary Table 3). Plasma MIP-1α decreased by 65% for both sexes at day 2 post-injury. Despite a gradual return to pre-injury ranges in males, MIP-1α levels remained below baseline at day 8 (1.8-fold; *p* = 0.018), day 17 (2.6-fold; *p* = 0.001) and day 31 (1.9-fold; *p* = 0.002) post-injury in females (Figure 2B and Supplementary Table 3). Similarly, while plasma MCP-1 remained comparable to pre-injury levels in males following ACL rupture, concentrations fell below baseline in females at day 17 (1.2-fold) and day 31 (1.4-fold), however these changes were not statistically significant (Supplementary Table 3). Plasma levels of the chemokine RANTES fell in both sexes, and remained below baseline to day 31 post-injury (Supplementary Table 3). No significant sex differences occurred for plasma IL-12 concentrations across the experimental period. However, while levels remained relatively stable in males, plasma IL-12 decreased 2.2-fold in females at day 2, followed by a gradual return to baseline thereafter (Supplementary Table 3). IFN-γ levels fell by ∼80% for both males and females at day 2 post-injury, with a return toward baseline over the remaining 29 days (Supplementary Table 3). Plasma TNF-α, IL-2 and IL-13 levels remained below the assay detection limit for both sexes throughout the experimental period (Supplementary Table 3).

### Sex differences in reparative responses in the knee

#### Joint function and macroscopic changes

**FIGURE 3:**
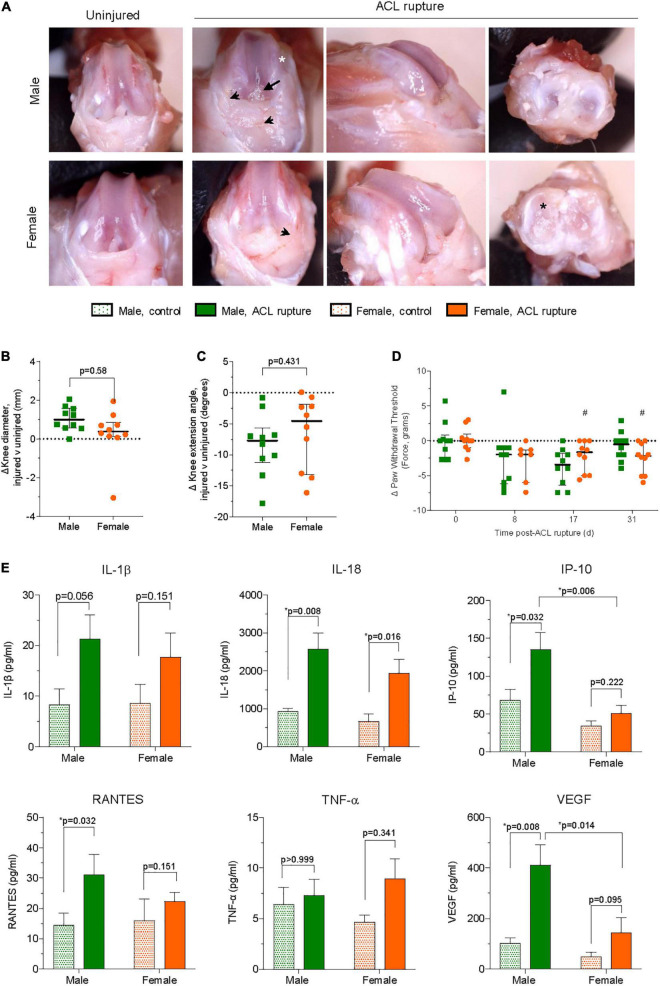
**(A)** Representative images of macroscopic changes in ACL-ruptured knees of male and female animals at day 31 post-injury. Synovial and capsular hyperplasia (arrowhead), articular cartilage defects on medial femoral condyles (white asterisk) and medial meniscus (black asterisk), and outgrowth of synovial tissue around ACL remnants (arrow) were frequently observed. Differences in **(B)** knee diameter, **(C)** knee extension angle, and **(D)** pain behavior between ACL-ruptured and control knees of male and female animals. Data show median ± IQR. Mann-Whitney *U*-test, ^#^*p* < 0.05 female, compared to baseline. **(E)** Synovial fluid concentrations of cytokines and chemokines in ACL-ruptured and control knees of male and female animals at day 31 post-injury. IL, interleukin; IP, interferon-inducible protein; RANTES, regulated upon activation, normal T cell expressed and presumably secreted; TNF, tumor necrosis factor; VEGF, vascular endothelial growth factor. Data show mean ± SEM. Two-way ANOVA, Sidak’s multiple comparisons test, *p < 0.05.

Anterior drawer test remained positive for all animals after 31 days. Following ACL rupture, joint swelling peaked at day 2 in both male and female animals (*p* < 0.001; Supplementary Figure 1). However, while swelling subsided within the first week of injury, differences in knee diameter of injured and contralateral control knees persisted for males at day 31 (*p* = 0.001) following ACL rupture which, upon dissection were attributed to joint capsule hyperplasia rather than joint effusion, *per se* (see below) (Figure 3B). Compared to control knees, knee extension angles were reduced in ACL-ruptured knees throughout the experimental period, with no significant sex differences observed (Supplementary Figure 1). At day 31 post-injury, the average loss of knee extension range of motion was 8.4° and 6.4° for male and female rats, respectively (*p* > 0.05; Figure 3C). Paw withdrawal threshold, an indicator of increased sensitivity to mechanical stimuli, was reduced in both sexes at day 17 (males, *p* = 0.057; females, *p* = 0.024), and in females at day 31 (*p* = 0.033) post-injury, suggesting persistence of mechanical hyperalgesia (Figure 3D). Upon dissection, mild-to-moderate effusion, synovial and capsular tissue hyperplasia, and articular cartilage defects were evident in ACL-ruptured knees at day 31 post-injury (Figure 3A), however no sex differences were observed in the macroscopic scores (Supplementary Figure 1). Outgrowth of synovial tissue around the tibial ACL remnant, and frequently involving attachment to the posterior cruciate ligament (PCL), was observed in both male and female animals (Figure 3A).

#### Inflammatory profile of synovial fluid

Compared to uninjured contralateral knees IL-1β and IL-18 levels were 2–2.5-fold higher, respectively in the synovial fluid of ACL-ruptured knees of both sexes at day 31 post-injury (Figure 3E). Elevations in IL-18 were significant for both males and females (*p* = 0.008 and *p* = 0.016, respectively). In contrast, IP-10 (CXCL10) was increased in male knees only following ACL rupture (*p* = 0.006; Figure 3E). Similarly, RANTES was significantly higher in ACL-ruptured than control knees of males (3-fold increase, *p* = 0.032), with smaller non-significant changes observed for females, and no difference between sexes (Figure 3E). Levels of TNF-α and MIP-1α were similar between control and ACL-ruptured male knees, however, 2- and 1.7-fold increases were observed in females 31-days following ACL injury (*p* > 0.05; Supplementary Table 4). Compared to control knees, levels of VEGF were increased for males (4-fold; *p* = 0.008) and females (2.5-fold; *p* = 0.095), with the response in males significantly greater than females following ACL rupture (*p* = 0.014; Figure 3E). Synovial IL-4 was 2.9- and 2-fold lower in ACL-ruptured compared to control knees of males (*p* = 0.064) and females (*p* = 0.079), respectively. No significant differences were observed in IL-17 levels between control and ACL-ruptured knees of either sex (Supplementary Table 4). Levels of IL-6, IL-1α, IL-10, IL-2, IFN-γ, IL-12p70, IL-13, GM-CSF, G-CSF, GRO/KC, and LIX were below the limit of detection in the synovial fluid at 31-days (Supplementary Table 4).

#### Inflammation and extracellular matrix remodeling within the anterior cruciate ligament remnant

**FIGURE 4:**
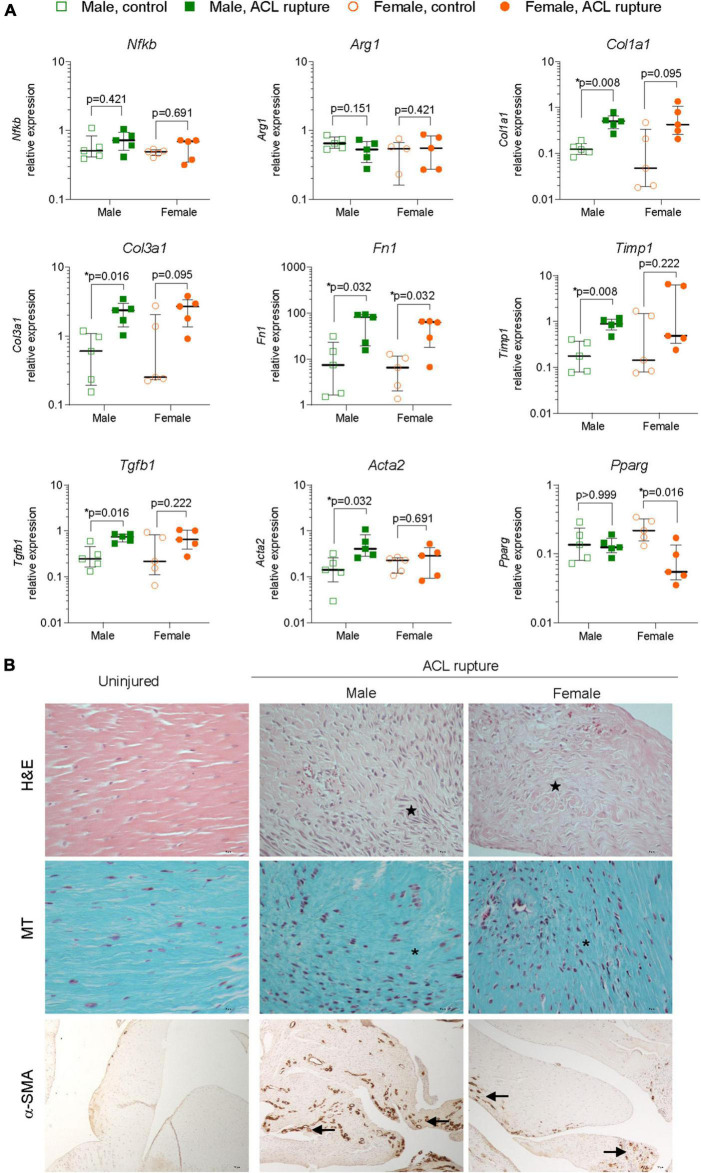
Inflammation and extracellular matrix (ECM) remodeling in the ACL remnant. **(A)** Relative expression of markers of inflammation (nuclear factor kappa B, Nfkb; arginase 1, Arg1); ECM components (collagen type 1, Col1a1; collagen type 3, Col3a1; fibronectin, Fn1); ECM remodeling enzymes (tissue inhibitor of matrix metalloproteinase 1, Timp1); and myofibroblast differentiation (transforming growth factor beta 1, Tgfb1; alpha smooth muscle actin, Acta2; peroxisome proliferator activated receptor gamma, Pparg) in the ACL remnant of male and female animals at day 31 post-injury. Data show median ± IQR. Mann-Whitney *U*-test, *p < 0.05. **(B)** Representative images of hematoxylin and eosin (H&E, magnification, 400×), Masson-Goldner trichrome (MT, magnification, ×400) and α-SMA (magnification, 400×) stained sections of the ACL remnant in control and injured knees of male and female animals. H&E-stained sections show loss of uniformity (star) in tissue architecture in ACL-ruptured, compared to control knees. MT staining shows disorganized collagen deposition in ACL-ruptured knees (asterisk), together with increased cellularity (collagen, blue; nuclei, pink). Positive α-SMA staining was associated with increased angiogenesis (arrow) in ACL remnants and adherent synovial tissue, and tended to be lower in female than male injured knees.

Gene expression levels of key inflammatory markers, core constituents of the ECM and myofibroblast differentiation were compared in ACL remnants of injured and contralateral control knees 31-days after ACL rupture. Small increases were observed in the expression of the key driver of inflammation, Nfkb, of injured vs. uninjured knees at day 31, however, changes were not statistically significant, nor was there any sex differences (Figure 4A). Similarly, no sex-specific differences were observed in expression of the M2 macrophage marker, Arg1 (Figure 4A). Expression of collagen types 1 and 3 increased in males (*p* = 0.008 and *p* = 0.016, respectively) and females (*p* = 0.095 and *p* = 0.095, respectively) relative to the uninjured knee (Figure 4A). Similarly, expression of Fn1 increased in the ACL remnant for both sexes (*p* = 0.032 for both; Figure 4A). Compared to uninjured knees, minor increases occurred in elastin Eln and Ccn2 for males, and Eln and Acan in the injured knees of females, however, these differences were not statistically significant (Supplementary Figure 2). The overall increase in gene expression of core ECM components was supported histologically, with loss of typical tissue architecture within the ACL, increased cellularity, and collagen deposition in irregular, dense and disorganized fiber arrangements (Figure 4B). No significant changes were observed in the expression of key ECM degradative enzymes, Mmp9 or Adamts4 in the ACL remnant for either sex (Supplementary Figure 2). Compared to the uninjured knee, expression of Timp1, an inhibitor of ECM degradative enzymes, increased significantly ∼4-fold in the ACL remnant in males, with no change observed in females (Figure 4A).

Myofibroblast differentiation and proliferation is a key event in tissue repair and is characterized by increased expression of Tgfb1, and Acta2, the gene for α-SMA, and is countered by the expression of the gene encoding Pparg nuclear receptor. Expression of both Tgfb1 and Acta2 were increased 3-fold in the ACL remnant in males, compared to control knees (*p* = 0.016 and *p* = 0.032, respectively) (Figure 4A). In females, Tgfb1 was also increased ∼3-fold, however, the increase was not significant, while Acta2 expression was similar between injured and uninjured knees (Figure 4A). α-SMA-positive immunohistochemical staining was higher in males than females and corresponded to increased angiogenesis in ACL remnants of injured knees at day 31 post-injury (Figure 4B). Interestingly, Pparg expression was 80% lower in the ACL remnant of injured knees in females compared to control knees (*p* = 0.016), with no change in males (Figure 4A).

#### Inflammation and extracellular matrix remodeling in the infrapatellar fat pad

**FIGURE 5:**
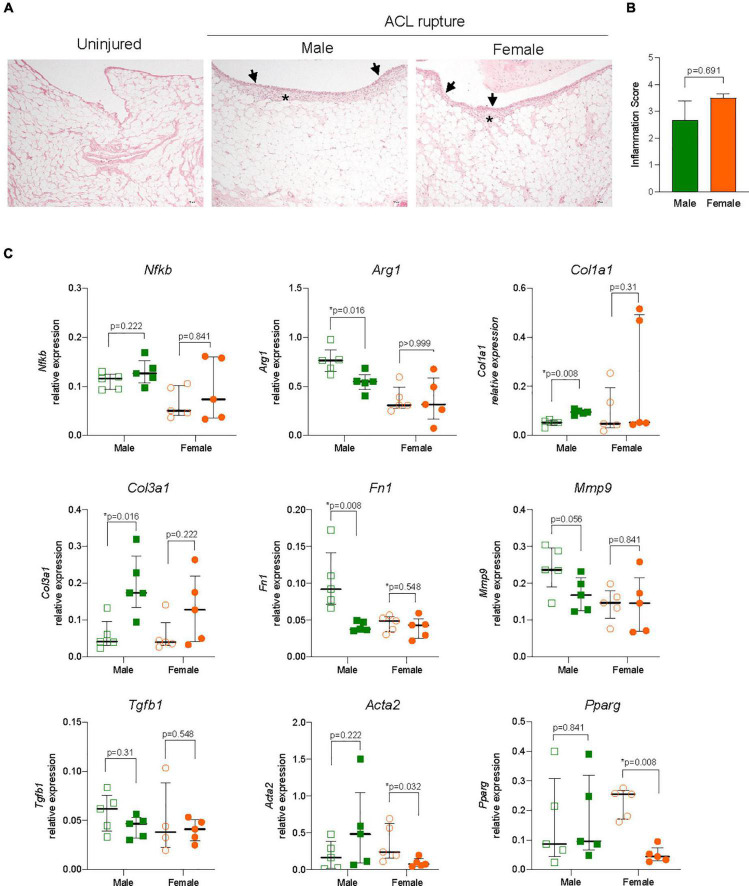
Inflammation and extracellular matrix (ECM) remodeling in the infrapatellar fat pad (IFP). **(A)** Representative hematoxylin and eosin (H&E) stained sections, and **(B)** inflammation score of the IFP showing inflammatory cell infiltration to the synovial lining (arrowheads) and sub-synovial tissue (asterisk) (magnification, 100×) from male and female animals. **(C)** Relative expression of markers of inflammation (nuclear factor kappa B, Nfkb; arginase 1, Arg1); ECM components (collagen type 1, Col1a1; collagen type 3, Col3a1; fibronectin, Fn1); ECM remodeling enzymes (matrix metalloproteinase 9, Mmp9); and myofibroblast differentiation (transforming growth factor beta 1, Tgfb1; alpha smooth muscle actin, Acta2; peroxisome proliferator activated receptor gamma, Pparg) within the IFP of ACL-ruptured and control knees of male and female animals. Data show median ± IQR. Mann-Whitney *U*-test, *p < 0.05.

The IFP plays an important role in local inflammatory and immune responses and fibrotic changes following ACL rupture. Synovitis was evident histologically with increased cellular infiltration within the synovial lining and sub-synovial tissue of IFP of ACL-ruptured knees compared to control knees (Figure 5A), without significant sex differences observed in inflammatory scores (Figure 5B) or Nfkb expression 31-days post-rupture (Figure 5C). However, a 35% reduction in Arg1 expression occurred in IFP from male ACL-ruptured knees (*p* = 0.016), with no change observed for females (Figure 5C). Similarly, while collagen expression was comparable in IFP from injured and control knees in females, Col1a1 and Col3a1 increased significantly in males following ACL rupture (*p* = 0.008 and *p* = 0.016, respectively) (Figure 5C). Compared to control knees, Fn1 expression decreased significantly in males only (*p* = 0.008; Figure 5C). No changes were found in expression levels of Acan, Eln, or Ccn2 in the IFP for either sex following injury (Supplementary Figure 2). Compared to control knees, Mmp9 expression decreased by 25% in the IFP of males (*p* = 0.056), with no change in females (Figure 5C). Expression levels of Adamts4, Timp1 and Tgfb1 were comparable in IFP of ACL-ruptured and control knees for both sexes (Figure 5C and Supplementary Figure 2). In contrast to the findings in the ACL remnant, Acta2 levels in injured vs. control knees did not change in males, whereas expression was 75% lower in females (*p* = 0.032). Pparg levels in the IFP from injured vs. control knees mirrored those observed in the ACL remnant, with no change in males but a significant 90% reduction in females (Figure 5C).

#### Inflammation and extracellular matrix remodeling in articular cartilage

**FIGURE 6:**
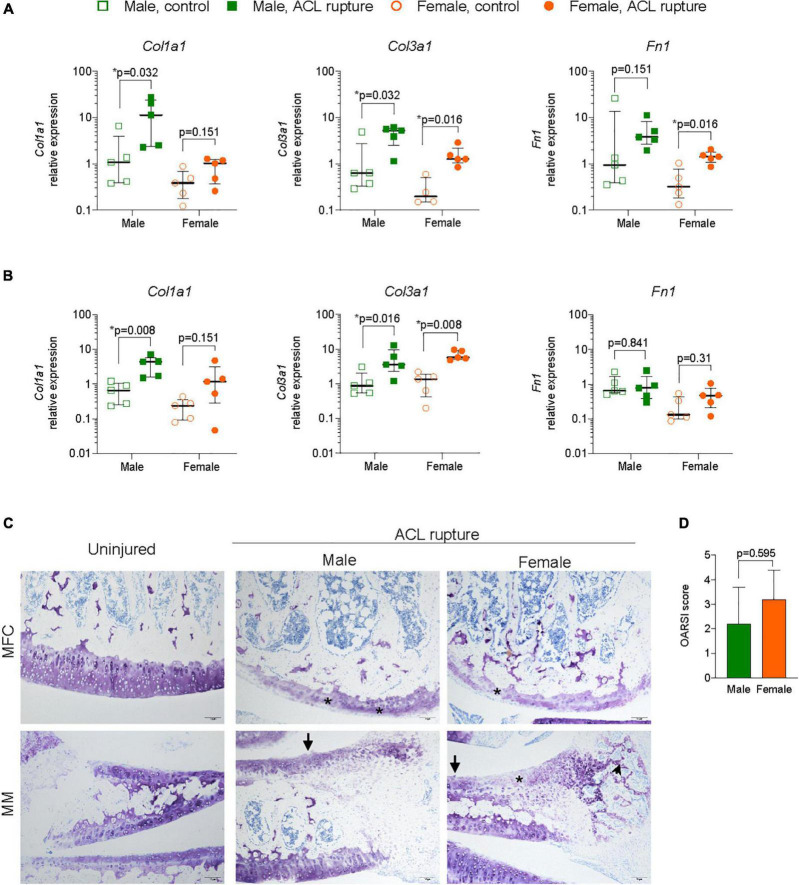
Extacellular matrix (ECM) remodeling in articular cartilage. Relative expression of collagen type 1 and 3 (Col1a1, Col3a1), and fibronectin (Fn1) in **(A)** medial femoral condylar (MFC) cartilage, and **(B)** medial meniscus (MM) of male and female animals at day 31 following ACL rupture. **(C)** Representative toluidine blue-stained sections of MFC (upper panels) and MM (lower panels) in uninjured and ACL-ruptured knees (magnification, 100×). Proteoglycan loss (asterisk) and surface fibrillation (arrows) was evident in MFC and MM, while disorganized ECM deposition was increased in the outer zone of the MM (arrowhead) of injured knees (magnification, 100×). **(D)** Osteoarthritis Research Society International (OARSI) scoring of medial articular cartilage surfaces in male and female animals at day 31 following ACL rupture. Data show median ± IQR. Mann-Whitney *U*-test, *p < 0.05.

Since ACL rupture increases the risk of degenerative changes within articular cartilage, particularly the medial compartments, expression levels of inflammatory markers, ECM components, remodeling enzymes and myofibroblast differentiation were assessed in cartilage from the MFC and MM of ACL-ruptured and control knees 31-days post-rupture. No significant sex differences, or differences between injured and control knees were found in expression of Nfkb, Arg1, or Eln in the MFC or MM (Supplementary Figures 3, 4). Col1a1 expression increased significantly in the MFC and MM of males after injury (*p* = 0.032 and *p* = 0.008, respectively), however changes in females were not statistically significant (Figures 6A,B). Col3a1 expression increased 10-fold in MFC and MM for both sexes (Figures 6A,B). Expression of Fn1 increased 6-fold in the MFC in both sexes (males, *p* = 0.151; females, *p* = 0.016), with no significant change in the MM for either sex (Figures 6A,B). While no changes were found in Acan expression in the MFC for either sex, there was a 60% decrease in Acan expression in MM for males (*p* = 0.056), with no change in females (Supplementary Figures 3, 4). In contrast, Ccn2 expression significantly increased 1.5-fold in the MFC of females, with no change in males between the injured and uninjured knee (Figure 6A). In the MM, expression levels of Ccn2 remained comparable in injured and uninjured knees of both sexes (Supplementary Figure 4). No significant changes were observed in expression of key ECM degradative enzymes Mmp9 or Adamts4 in the MFC or MM (Supplementary Figures 3, 4). In contrast, inhibitor of ECM degradation, Timp1, was increased in both the MFC (males, *p* = 0.151; females, *p* = 0.032) and MM (males, *p* = 0.008; females, *p* = 0.008) in both sexes following injury (Supplementary Figures 3, 4). No changes were found in Tgfb1 or Acta2 expression in the MFC (Supplementary Figure 4). In the MM, Tgfb1 expression increased 2-fold in both sexes following injury (*p* = 0.056), though no differences were found in Acta2 expression (Figure 6B and Supplementary Figure 4). Histologically, mild degenerative changes were visualized in the cartilage of the MFC and MM of injured and control knees of males and females at day 31 following ACL rupture, with OARSI scores comparable for males and females (Figures 6C,D). There was evidence of minor proteoglycan loss and focal surface fibrillation in the MFC of injured knees for both sexes. Increased cellularity and disorganized collagen deposition was observed in the outer zone of the MM in ACL-ruptured knees, however, changes were comparable between sexes.

## Discussion

The effect of sex on joint tissue repair processes following an ACL rupture are poorly understood (2). We report in a rat model of non-surgical ACL rupture: (1) both sexes showed similar functional responses early after ACL rupture, however, females had persistent mechanical hyperalgesia; (2) males and females showed different hematological and systemic inflammatory responses over 31-days; (3) males had significantly higher levels of VEGF, RANTES and IP-10 chemokines in synovial fluid compared to females; (4) Acta2 gene expression (α-SMA production) in the ACL remnant was significantly higher in males with no change in females; and (5) sex differences in ECM remodeling were also apparent in the IFP and articular cartilage. These sex differences will now be discussed.

### Systemic inflammatory response

In the first few days after ACL rupture, we found changes in the timing, direction and magnitude of circulating lymphocyte, monocyte and neutrophil counts in males and females. These early hematological changes were accompanied by changes in plasma cytokines, which appeared to set the stage for the sex-specific differences in joint tissue healing observed at 31-days. For example, the fall in monocytes in females at 2 days post-injury was accompanied by a 70% decrease in pro-inflammatory IL-1β, a 54% decrease in IL-12, and a small ∼20% increase in anti-inflammatory IL-4, whereas in males IL-1β increased by ∼20%, IL-12 did not change and IL-4 fell by 84%. Since IL-1β and IL-12 are produced by activated monocytes and macrophages (19), their lower plasma levels in females may suggest increased uptake of these inflammatory cells into the joint (20, 21). Another intriguing result was that while plasma IL-α remained below or comparable to preinjury levels in males throughout the experimental period, minor increases occurred in females at day 8 and day 31 after ACL rupture. IL-1α is constitutively expressed by many cell types and, in contrast to IL-1β, can act as a damage-associated molecular pattern (DAMP) to enhance inflammatory cell recruitment after tissue injury (22, 23). Thus, the sex differences in plasma IL-1α and IL-1β may reflect differences in the timing of repair responses within the joint, and are supported by contrasting temporal changes in monocyte and neutrophil numbers within the first 8-days following ACL rupture. Sex-specific opposing responses also occurred for IL-4 and IL-10 at day 2 and 8 post-injury. In females, an increase in IL-4 was accompanied by a decrease in IL-10, while a reduction in IL-4 in males was accompanied by a 6-fold increase in IL-10. Interestingly, sexual dimorphism was recently described for IL-10’s anti-inflammatory action toward leukocytes, and for IL-4-induced monocyte polarization (24, 25). It is possible that the contrasting plasma cytokine responses we observed reflect differences in the cytokine responsiveness of leukocytes in males and females, and that this may underlie subtle changes in the pattern of inflammatory cell mobilization and recruitment to the site of injury.

### Persistent joint inflammation

As anticipated from the systemic changes, sex differences in inflammatory mediators were found in the synovial fluid of injured knees, 31-days after ACL rupture. Despite both sexes having similar increases in inflammasome-activators IL-1β and IL-18 (26), we found significant increases in three major chemokines VEGF, RANTES and IP-10 in males, with little or no change in females. Higher VEGF levels indicate a heightened inflammatory and angiogenic potential in the synovial fluid (27–29); and higher RANTES and IP-10 indicate a greater number of effector and regulatory T lymphocytes, natural killer T cells (NKT), and NK cells within the injured tissues of males compared to females (30). Another contrasting sex difference in the synovial fluid was the increase in MIP-1α, a critical macrophage chemoattractant in wound healing (31) in female injured knees, compared to males. We conclude the synovial fluid of both sexes displayed different inflammatory environments with no apparent signs of subsiding after 31 days. Unfortunately, we did not characterize the different populations of immune cells in the synovial fluid at different times after ACL rupture to further investigate these responses.

### Joint tissue repair and remodeling

Our study also examined sex-specific reparative processes within the ACL-ruptured knee compared to the contralateral knee. The ACL remnant (and adjacent tissues) are the main sources of DAMPs, cytokines, chemokines, proteases, pain and neural signals that are communicated between the joint, blood and central nervous system (CNS) (32–34). Different signals during healing lead to different responses with most ACL remnants failing to self-heal and restore normal joint function. When wound repair is completed, the activity of Tgfb normally returns to baseline. In our study, increases in Tgfb gene expression and up-to-20-fold increases in type 1 and 3 collagens and fibronectin expression indicate ECM remodeling was ongoing in both sexes at day 31 (35). The deposition and expansion of the ECM was supported histologically, with the loss of normal tissue architecture, disorganized collagen fiber orientation, chondroid metaplasia and increased cellularity in both sexes.

However, sex differences were found in ECM remodeling. Male remnants appeared to have a higher potential for differentiation of fibroblasts to myofibroblasts with higher tissue contractile elements (stiffness) than females. This observation was based on the 4-fold higher Acta2 expression and α-SMA production in males with no change in females (35–37). A higher stiffness component of stress fibers in males was further supported by the little or no change in Eln. Eln codes for tropoelastin, which is a spring-like molecule that can stretch up to eight times its resting length and when attached together forms the elastin part of the ligament (38). While the production of α-SMA by myofibroblasts and resistance to apoptosis is a hallmark of pathological fibrosis (35, 37, 39), based on this data it is premature at 31 days to conclude increased fibrosis in males.

The second line of evidence for reduced fibroblast differentiation in females, comes indirectly from sex differences in Pparg expression. Male ACL remnants showed no change in Pparg whereas females had significantly reduced expression by 80%. In many cell types, Pparg downregulation has been shown to decrease cell differentiation (40, 41), and the 80% fall in females may indicate downregulation of fibroblast to myofibroblast differentiation. However, few studies have examined the role of Pparg in repair of ligaments, and Pparg has other functions including cell proliferation, differentiation, modulating adipogenesis, inflammation and fibrosis (42, 43). Further studies are required to characterize the role of Pparg following ACL rupture.

Sex-specific differences were also apparent in the articular tissues and IFP of the injured knee after ACL rupture. ECM remodeling in MFC, MM, and IFP, for example, involved significant increases in collagen type 1 expression in males with little or no change in females, whereas collagen type 3 significantly increased in both MFC and MM, but not in the IFP. The increase in collagen type 1 in MFC and MM of males at 31 days is noteworthy and may indicate the development of pathological cartilage (44–46). In normal hyaline cartilage, the predominant collagen type is type 2, however, during early fibrosis and osteoarthritis, de-differentiated chondrocytes can switch from producing collagen type 2 to collagen type 1 (44–46). Type 1 collagen can also be produced by fibroblasts during matrix building (44–46). Increased collagen type 1 in males may therefore be an early signature of fibrosis or early post-traumatic osteoarthritis (PTOA) compared to females, preceding histologically apparent differences since we found comparable mild degenerative changes for both sexes at day 31 post-injury.

ECM remodeling differences were also found in fibronectin expression with 6-fold increases in MFC, small or no increases in MM, and 50% decreases in the IFP of males, with no change in females. How these changes relate to collagen differences are not known. Another curious result, and similar to the ACL remnant, was the profound 90% decrease in expression of cellular differentiation marker Pparg in the IFP of females with little change in males. Since the IFP is a source of perivascular stem cells, a decrease of Pparg expression here may reflect a reduction in cellular differentiation requirement in female IFP compared to males at this time (40, 47). In summary, we showed that ACL rupture leads to significant injury and remodeling in adjacent tissues, and that sex-specific differences may impact on healing of the whole joint.

### Clinical significance

The clinical significance of our study relates to filling a knowledge gap on sex-specific healing patterns after ACL rupture with no intervention. For many years, it was believed that an ACL tear or rupture could not repair itself. Subsequent studies have shown a ruptured ACL possesses some regenerative capacity, however, it is insufficient to sustain the compression and tension loads required for normal joint function (48–51). In addition, our study shows ACL rupture is not an isolated injury but affects the whole joint, and that significant sex differences occur during tissue repair. This is the first study of a larger body of continuing work evaluating the effect of ACLR surgery carried out at day 3 and 14 after rupture, on sex-specific outcomes and underlying mechanisms of healing. The present finding that males have a higher stiffness component and increased angiogenesis compared to females implies healing may take longer in males, with increased potential for PTOA. Sex-differences in myofibroblast activation and angiogenesis following myocarditis and ischemic injury have also been reported, with preponderance for upregulation of ECM and fibrosis-related pathways in males, than females (52–55). Future studies will examine the effect of early and delayed surgical intervention on the disparate healing responses found between sexes.

### Limitations

We acknowledge that ACL injury may cause subtle changes in hind-limb weight-bearing and gait in rodents, as it would in humans, which may induce responses in both the injured and uninjured contralateral limb. For this reason, we reported inflammatory and healing responses of the ACL-ruptured knee relative to the contralateral limb, rather than a naive control group. A further limitation of the present study was we did not perform immunoprofiling analyses to identify changes in the number and type of inflammatory and immune cells in the different compartments (systemic, synovial fluid and joint tissues) at earlier and later time points. In addition, electron microscopy may shed further light on potential sex differences in the ultrastructural ECM morphology in joint tissues in response to ACL rupture.

## Conclusion

We conclude that ACL rupture affects the entire joint and sex differences in the healing response were evident in the circulation, synovial fluid and joint tissues. Although inflammatory-based ACL remodeling persisted in both sexes at 31 days, males appeared to show greater myofibroblast-driven ECM deposition and expansion with higher tissue contractile elements than females. It is currently not known if these sex-specific differences could be useful in selection criteria for timing of surgery, or if they continue to impact on healing following ACLR surgery.

## Data availability statement

All data pertaining to this study have been included in this manuscript and Supplementary material.

## Ethics statement

The animal study was reviewed and approved by the James Cook University Animal Ethics Committee, and the US Animal Care and Review Use Office.

## Author contributions

PM, GD, and JM: concept. GD, JM, and HL: experimental design. JM, HL, and EB: data collection. GD and JM: data analyses, interpretation, and manuscript preparation. PM, HL, and EB: editing. All authors contributed to the article and approved the submitted version.
