# Supplementary material for: Female rats have a different healing phenotype than males after anterior cruciate ligament rupture with no intervention
Source: Front Med (Lausanne). 2022 Nov 14;9:976980. doi: 10.3389/fmed.2022.976980 (PMC9701729; doi:10.3389/fmed.2022.976980)
Supplement: Supplementary file 1 [file Data_Sheet_1.PDF]

Table S1. Primers used for gene expression analysis.

Gene	QuantiTect Primer Assay (Qiagen)
Acan	QT00189518
Acta2	QT01615901
Adamts4	QT02475851
Arg1	QT00177611
Ccn2	QT00182021
Col1a1	QT00366016
Col3a1	QT00365981
Eln	QT00413007
Fn1	QT00179333
Hprt1	QT00199640
Mmp9	QT00178290
Nfkb1	QT00370545
Pparg	QT00186172
Tgfb1	QT00187796
Timp1	QT00185304

Acan, aggrecan; *Acta2,* actin alpha 2, smooth muscle; *Adamts4,* ADAM metallopeptidase with thrombospondin type 1 motif, 4; *Arg1,* arginase 1; *Ccn2,* cellular communication network factor 2; *Col1a1,* collagen type I alpha 1 chain; *Col3a1,* collagen type III alpha 1 chain; *Eln,* elastin; *Fn1,* fibronectin 1; *Hprt1,* hypoxanthine phosphoribosyltransferase 1 (housekeeping gene); *Mmp9,* matrix metallopeptidase 9; *Nfkb1,* nuclear factor kappa B subunit 1; *Pparg,* peroxisome proliferator-activated receptor gamma; *Tgfb1,* transforming growth factor, beta 1; *Timp1,* TIMP metallopeptidase inhibitor 1
