# Supplementary material for: Female rats have a different healing phenotype than males after anterior cruciate ligament rupture with no intervention
Source: Front Med (Lausanne). 2022 Nov 14;9:976980. doi: 10.3389/fmed.2022.976980 (PMC9701729; doi:10.3389/fmed.2022.976980)
Supplement: Supplementary file 2 [file Data_Sheet_2.PDF]

Table S2. Post-ACL rupture hematology for male (n=10) and female (n=10) animals.

**Indices**	Time	Male	Female	p value
WBC, x 10^9cells/L	Baseline	11.2 ± 1.9	10.7 ± 3.5	0.999
	day 2	10.8 ± 4.7	9.5 ± 5.3	0.998
	day 4	9.2 ± 3.8	11.2 ± 4.6	0.917
	day 6	13.0 ± 4.1	10.3 ± 3.8	0.700
	day 8	8.7 ± 3.3	10.8 ± 3.8	0.807
	day 17	9.7 ± 3.1	10.0 ± 4.9	>0.999
	day 31	10.0 ± 3.9	8.1 ± 4.0	0.907
Lymphocytes, x 10^9 cells/L	Baseline	9.1 ± 1.5	8.3 ± 2.6	0.978
	day 2	7.8 ± 2.9	7.2 ± 4.5	0.999
	day 4	6.9 ±2.9	7.7 ± 2.7	0.998
	day 6	9.3 ± 2.3	7.3 ± 2.7	0.545
	day 8	6.9 ± 2.5	7.1 ± 2.2	>0.999
	day 17	7.1 ± 2.1	6.9 ± 3.1	>0.999
	day 31	7.5 ± 3.0	5.9 ± 2.6	0.826
Lymphocytes, %	Baseline	81.5 ± 5.7	78.0 ± 5.7	0.768
	day 2	74.5 ± 6.7	75.7 ± 8.8	>0.999
	day 4	75.6 ± 6.4	70.1 ± 9.0	0.624
	day 6	72.8 ± 7.7	71.1 ± 8.3	0.999
	day 8	78.5 ± 4.7	65.7 ± 9.7[#]	0.016*
	day 17	74.0 ± 5.2	70.7 ± 10.5	0.969
	day 31	74.6 ± 5.5	76.0 ±8.4	0.999
Monocytes, x 10^9cells/L	Baseline	0.7 ± 0.7	0.7 ± 0.3	>0.999
	day 2	0.7 ± 0.9	0.5 ± 0.4	0.990
	day 4	0.1 ± 0.2	0.8 ± 0.8	0.156
	day 6	1.1 ± 1.0	0.6 ± 0.5	0.883

	day 8	0.2 ± 0.4	1.0 ± 0.8	0.058
	day 17	0.7 ± 0.7	0.9 ± 0.7	0.985
	day 31	0.8 ± 0.5	0.5 ± 0.5	0.919
Monocytes, %	Baseline	5.7 ± 5.5	6.4 ± 3.5	>0.999
	day 2	5.0 ± 5.4	5.2 ± 4.3	>0.999
	day 4	1.8 ± 2.6	5.8 ± 4.5	0.186
	day 6	7.1 ± 5.8	5.6 ± 3.6	0.992
	day 8	1.6 ± 2.2	8.2 ± 4.6	0.009*
	day 17	5.8 ± 5.2	8.9 ± 4.1	0.698
	day 31	8.5 ± 5.0	5.2 ± 3.7	0.579
Neutrophils	Baseline	1.5 ± 0.6	1.7 ± 1.0	0.984
x 10^9cells/L	day 2	2.2 ± 1.1	1.8 ± 1.0	0.965
	day 4	2.1 ± 1.0	2.8 ± 1.5	0.887
	day 6	2.6 ± 1.1^	2.4 ± 1.0	0.998
	day 8	1.7 ± 0.5	2.7 ± 1.2	0.213
	day 17	1.9 ± 0.7	2.1 ± 1.6	0.999
	day 31	1.7 ± 0.9	1.6 ± 1.2	>0.999
Neutrophils, %	Baseline	12.8 ± 4.3	15.6 ± 5.8	0.842
	day 2	20.5 ± 2.5^	19.2 ± 5.8	0.995
	day 4	22.6 ± 4.1^	24.1 ± 7.1	0.997
	day 6	20.1 ± 3.2^	23.1 ± 5.2	0.674
	day 8	19.9 ± 4.8^	26.1 ±7.2$^{\#}$	0.236
	day 17	20.1 ± 3.9^	20.3 ± 7.3	>0.999
	day 31	17.0 ± 3.9	18.7 ± 6.9	0.991
NLR	Baseline	0.16 ± 0.06	0.21 ± 0.9	0.780
	day 2	0.28 ± 0.05^	0.26 ± 0.11	>0.999
	day 4	0.31 ± 0.09	0.36 ± 0.15	0.948

	day 6	0.28 ± 0.07^	0.34 ± 0.12	0.844
	day 8	0.26 ± 0.08	0.4 ±0.16#	0.171
	day 17	0.28± 0.07	0.31 ± 0.16	0.997
	day 31	0.23 ± 0.07	0.26 ± 0.12	0.995
Platelets, x 10^9/L	Baseline	562 ± 141	322 ± 254	0.134
	day 2	364 ± 191	324 ± 201	0.999
	day 4	481 ± 202	283 ± 204	0.261
	day 6	340 ± 220	300 ± 242	0.999
	day 8	465 ± 160	228 ± 160	0.027*
	day 17	346 ± 173^	283 ± 227	0.992
	day 31	360 ± 196	276 ± 189	0.947
RBC, x 10^{12}/L	Baseline	6.8 ± 0.3	6.7 ± 0.4	0.996
	day 2	7.7 ± 0.4^	7.3 ±0.8	0.864
	day 4	7.5 ± 0.3^	7.0 ± 0.5	0.084
	day 6	7.5 ± 0.2^	6.7 ± 0.3	<0.001*
	day 8	7.6 ± 0.3^	6.5 ± 0.3	<0.001*
	day 17	7.6 ± 0.3^	6.8 ± 0.5	0.011*
	day 31	8.0 ± 0.3^	7.3 ±0.3#	<0.001*
Hematocrit, %	Baseline	40.9 ± 2.1	40.9 ± 2.6	>0.999
	day 2	37.5 ± 2.2^	36.5 ± 3.5#	0.986
	day 4	36.6 ± 1.7^	35.0 ± 2.1#	0.416
	day 6	36.3 ± 1.5^	33.4 ± 1.4#	0.002*
	day 8	37.0 ± 1.5^	32.5 ± 1.9#	<0.001*
	day 17	36.6 ± 2.1^	34.8 ± 2.3#	0.422
	day 31	38.3 ±1.3^	37.3 ± 2.1#	0.818
Hemoglobin, g/dL	Baseline	13.1 ± 0.7	12.6 ± 0.9	0.792
	day 2	13.2 ± 0.7	13.1 ± 1.1	>0.999

	day 4	13.1 ± 0.7	12.3 ± 0.7	0.158
	day 6	12.9 ± 0.5	12.0 ± 0.5	0.005*
	day 8	13.0 ± 0.5	11.6 ± 0.8	0.002*
	day 17	12.9 ± 0.8	12.4 ± 0.8	0.713
	day 31	13.3 ± 0.6	13.3 ± 0.6	>0.999

Data show mean ± standard deviation. Baseline values were obtained from 10 healthy male and female Sprague-Dawley rats. RBC, red blood cell; WBC, white blood cell; NLR, neutrophil to lymphocyte ratio. Two-way ANOVA with Sidak's multiple comparisons test. * $p <0.05$, male compared to female; ^ $p < 0.05$ male, compared to baseline; # $p < 0.05$ female, compared to baseline.
