# Supplementary material for: Female rats have a different healing phenotype than males after anterior cruciate ligament rupture with no intervention
Source: Front Med (Lausanne). 2022 Nov 14;9:976980. doi: 10.3389/fmed.2022.976980 (PMC9701729; doi:10.3389/fmed.2022.976980)
Supplement: Supplementary file 3 [file Data_Sheet_3.PDF]

Table S3. Systemic inflammatory cytokine and chemokine response for male (n=10) and female (n=10) animals following ACL rupture.

**Analyte (pg/ml)**	Time	Male	Female	p value
IFN-γ	Baseline	264.0 ± 173.2	94.8 ± 73.5	0.071
	day 2	47.1 ± 80.0	14.5 ± 0.0	0.895
	day 8	202.9 ± 85.4	176.9 ± 257.3	0.999
	day 17	121.4 ± 121.1	116.3 ± 208.5	>0.999
	day 31	164.5 ± 80.0	170.3 ± 286.9	>0.999
IL-1α	Baseline	46.5 ± 29.7	29.5 ± 15.8	0.510
	day 2	40.1 ± 42.8	22.4 ± 23.4	0.923
	day 8	28.1 ± 17.2	57.4 ± 69.6	0.721
	day 17	25.1 ± 26.3	29.5 ± 36.6	0.999
	day 31	30.2 ± 9.8	64.4 ± 101.1	0.849
IL-1β	Baseline	14.6 ± 8.3	11.4 ± 7.8	0.912
	day 2	17.4 ± 29.4	3.6 ± 1.2	0.835
	day 8	68.7 ± 110.4	12.6 ± 12.7	0.600
	day 17	38.7 ± 58.5	7.9 ± 3.6	0.565
	day 31	54.9 ± 86.3	10.0 ± 11.2	0.579
IL-2	Baseline	n.d.	n.d.	-
	day 2	n.d.	n.d.	-
	day 8	n.d.	n.d.	-
	day 17	n.d.	n.d.	-
	day 31	n.d.	n.d.	-
IL-4	Baseline	79.0 ± 77.8	35.9 ± 30.9	0.501
	day 2	13.4 ± 14.7^	43.8 ± 62.5	0.824
	day 8	41.2 ± 19.0	98.0 ± 158.4	0.818
	day 17	24.6 ± 27.0	43.9 ± 70.9	0.946

	day 31	45.2 ± 15.0	40.8 ± 64.6	0.999
IL-6	Baseline	216.1 ± 143.7	120.0 ± 79.0	0.360
	day 2	73.1 ± 0.0	73.1 ± 0.0	>0.999
	day 8	138.9 ± 54.2	313.9 ± 378.3	0.631
	day 17	116.6 ± 68.4	115.8 ± 88.2	>0.999
	day 31	160.0 ± 71.5	253.0 ± 488.4	0.985
IL-10	Baseline	78.5 ± 29.5	57.0 ± 36.5	0.596
	day 2	42.6 ± 53.6	9.5 ± 5.5	0.655
	day 8	245.4 ± 351.2	44.0 ± 35.7	0.485
	day 17	144.0 ± 194.8	37.2 ± 14.0	0.527
	day 31	222.1 ± 329.7	47.4 ± 48.6	0.563
IL-12p70	Baseline	56.2 ± 21.6	52.1 ± 24.1	0.998
	day 2	56.5 ± 77.3	24.1 ± 28.5	0.902
	day 8	44.1 ± 35.4	55.7 ± 73.4	0.996
	day 17	34.9 ± 21.9^	41.8 ± 40.2	0.994
	day 31	43.9 ± 20.2	96.3 ± 155.8	0.853
IL-13	Baseline	n.d.	n.d.	-
	day 2	n.d.	n.d.	-
	day 8	n.d.	n.d.	-
	day 17	n.d.	n.d.	-
	day 31	n.d.	n.d.	-
MCP-1	Baseline	1087.6 ± 391.9	916.2 ± 170.4	0.645
	day 2	n.a.	n.a.	-
	day 8	884.5 ± 239.4	902.7 ± 625.1	>0.999
	day 17	1044.0 ± 201.6	734.5 ± 305.4	0.072
	day 31	963.3 ± 283.7	673.1 ± 321.2	0.217
MIP-1α	Baseline	15.5 ± 5.6	14.5 ± 4.8	0.996

	day 2	6.2 ± 5.7	5.0 ± 3.4	0.996
	day 8	9.0 ± 8.7	7.9 ± 4.7#	0.999
	day 17	10.8 ± 6.1	5.6 ± 3.3#	0.197
	day 31	11.9 ± 8.2	7.7 ± 5.3#	0.700
RANTES	Baseline	2071.1 ± 1490.1	1850.1 ± 928.2	0.997
	day 2	1326.6 ± 523.7	1378.3 ± 617.2	>0.999
	day 8	1253.4 ± 743.4	1951.3 ± 1762.4	0.799
	day 17	1630.6 ± 714.4	1035.5 ± 570.0	0.283
	day 31	1544.4 ± 685.8	1104.0 ± 813.3	0.707
TNF-α	Baseline	n.d.	n.d.	-
	day 2	n.d.	n.d.	-
	day 8	n.d.	n.d.	-
	day 17	n.d.	n.d.	-
	day 31	n.d.	n.d.	-

Data show mean ± standard deviation. Baseline values were obtained from 10 healthy male and female Sprague-Dawley rats. n.a., unable to be assessed due to low sample volume; n.d., not detected; IFN-γ, interferon gamma; IL, interleukin; MIP-1α, macrophage inflammatory protein-1 alpha; MCP-1, monocyte chemoattractant protein-1; RANTES, Regulated upon Activation, Normal T Cell Expressed and Presumably Secreted; TNF-α, tumor necrosis factor alpha. Assay limit of detection (pg/ml): IFN-γ, 14.6; IL-1α, 12.2; IL-1β, 2.8; IL-2, 12.2; IL-4, 4.9; IL-6, 73.2; IL-10, 2.7; IL-12p70, 12.2; IL-13, 4.9; MIP-1α, 2.4; RANTES, 2.5; MCP-1, 9.0; TNF-α, 2.1.Mixed-effects model with Sidak's multiple comparisons test. * $p < 0.05$, male compared to female; ^ $p < 0.05$ male, compared to baseline; # $p < 0.05$ female, compared to baseline.
