# Supplementary material for: Female rats have a different healing phenotype than males after anterior cruciate ligament rupture with no intervention
Source: Front Med (Lausanne). 2022 Nov 14;9:976980. doi: 10.3389/fmed.2022.976980 (PMC9701729; doi:10.3389/fmed.2022.976980)
Supplement: Supplementary file 4 [file Data_Sheet_4.PDF]

Table S4. Inflammatory cytokine and chemokine profile of synovial fluid for male (n=5) and female (n=5) animals at day 31 following ACL rupture.

**Analyte (pg/ml)**		Male	Female	p value
Fractalkine	ACL intact	10.2 ± 7.7	17.1 ± 14.3	0.845
	ACL rupture	15.1 ± 5.0	14.7 ± 8.5	>0.999
G-CSF	ACL intact	n.d.	n.d.	-
	ACL rupture	n.d.	n.d.	-
GM-CSF	ACL intact	n.d.	n.d.	-
	ACL rupture	n.d.	n.d.	-
GRO/KC	ACL intact	n.d.	n.d.	-
	ACL rupture	n.d.	n.d.	-
IFN-γ	ACL intact	n.d.	n.d.	-
	ACL rupture	n.d.	n.d.	-
IL-1α	ACL intact	n.d.	n.d.	-
	ACL rupture	n.d.	n.d.	-
IL-1β	ACL intact	8.3 ± 7.2	8.6 ± 8.4	>0.999
	ACL rupture	21.3 ± 10.6	17.7 ± 10.6	0.992
IL-2	ACL intact	n.d.	n.d.	-
	ACL rupture	n.d.	n.d.	-
IL-4	ACL intact	78.2 ± 52.5	128.7 ± 58.2	0.599
	ACL rupture	27.4 ± 17.6	63.4 ± 64.8	0.868
IL-6	ACL intact	n.d.	n.d.	-
	ACL rupture	n.d.	n.d.	-
IL-10	ACL intact	n.d.	n.d.	-
	ACL rupture	n.d.	n.d.	-
IL-12p70	ACL intact	n.d.	n.d.	-

	ACL rupture	n.d.	n.d.	-
IL-13	ACL intact	n.d.	n.d.	-
	ACL rupture	n.d.	n.d.	-
IL-17A	ACL intact	27.0 ± 26.1	23.2 ± 16.5	0.999
	ACL rupture	22.9 ± 16.7	21.3 ± 6.9	>0.999
IL-18	ACL intact	934.4 ± 175.7	658.8 ± 451.3	0.989
	ACL rupture	2571.7 ± 956.2^	1943.4 ± 804#	0.643
IP-10	ACL intact	68.2 ± 32.5	34.6 ± 13.5	0.551
	ACL rupture	135.1 ± 50.5^	51.0 ± 22.7	0.005*
LIX	ACL intact	n.d.	n.d.	-
	ACL rupture	n.d.	n.d.	-
MIP-1α	ACL intact	5.7 ± 3.7	3.1 ±0.8	0.65
	ACL rupture	6.8 ±3.3	5.4 ± 2.3	0.97
RANTES	ACL intact	14.4 ± 8.8	16.0 ± 16.0	>0.999
	ACL rupture	31.1 ± 15.1	22.3 ± 6.5	0.849
TNF-α	ACL intact	6.4 ± 3.7	4.7 ± 1.6	0.967
	ACL rupture	7.3 ± 3.5	9.0 ± 4.4	0.975
VEGF	ACL intact	102.1 ± 48.5	49.1 ± 40.3	0.981
	ACL rupture	410.0 ± 181.3^	144.8 ±132.5	0.014*

Data show mean ± standard deviation. n.d., not detected; G-CSF, granulocyte colony-stimulating factor; GM-CSF, granulocyte-macrophage colony-stimulating factor; GRO/KC, growth-regulated oncogene/keratinocyte chemoattractant; IFN-γ, interferon gamma; IL, interleukin; IP-10, interferon gamma-induced protein 10; LIX, lipopolysaccharide-induced CXC chemokine; MIP-1α, macrophage inflammatory protein-1 alpha; MCP-1, monocyte chemoattractant protein-1; RANTES, Regulated upon Activation, Normal T Cell Expressed and Presumably Secreted; TNF-α, tumor necrosis factor alpha; VEGF, vascular endothelial growth factor. Assay limit of detection (pg/ml): fractalkine, 1.5; G-CSF,4.9; GM-CSF, 12.2; GRO/KC, 14.6; IFN-γ, 14.6; IL-1α, 12.2; IL-1β, 2.8; IL-2, 12.2; IL-4, 4.9; IL-6, 73.2; IL-10, 2.7; IL-12p70, 12.2; IL-13, 4.9; IL-17A, 7.3; IL-18, 12.5; IP-10, 3.5; LIX, 24.4; MIP-1α, 2.4; RANTES, 2.5; TNF-α, 2.1; VEGF, 8.6. Mixed-effects model with Sidak's multiple comparisons test. * $p < 0.05$, male compared to female; ^ $p < 0.05$ male, rupture compared to intact; # $p < 0.05$ female, rupture compared to intact.
