# Supplementary material for: Female rats have a different healing phenotype than males after anterior cruciate ligament rupture with no intervention
Source: Front Med (Lausanne). 2022 Nov 14;9:976980. doi: 10.3389/fmed.2022.976980 (PMC9701729; doi:10.3389/fmed.2022.976980)

Figure S1. Changes in A) body weight, B) joint swelling, C) static knee extension angle and D) macroscopic pathology in male (green) and female (orange) animals following ACL rupture. Data show mean ± SEM. (median ± IQR for macroscopic scores). Two-way ANOVA, Sidak's multiple comparison's test (Mann-Whitney U test for macroscopic scores). Significance compared to baseline, ^ $p < 0.05$ male and # $p < 0.05$ female.

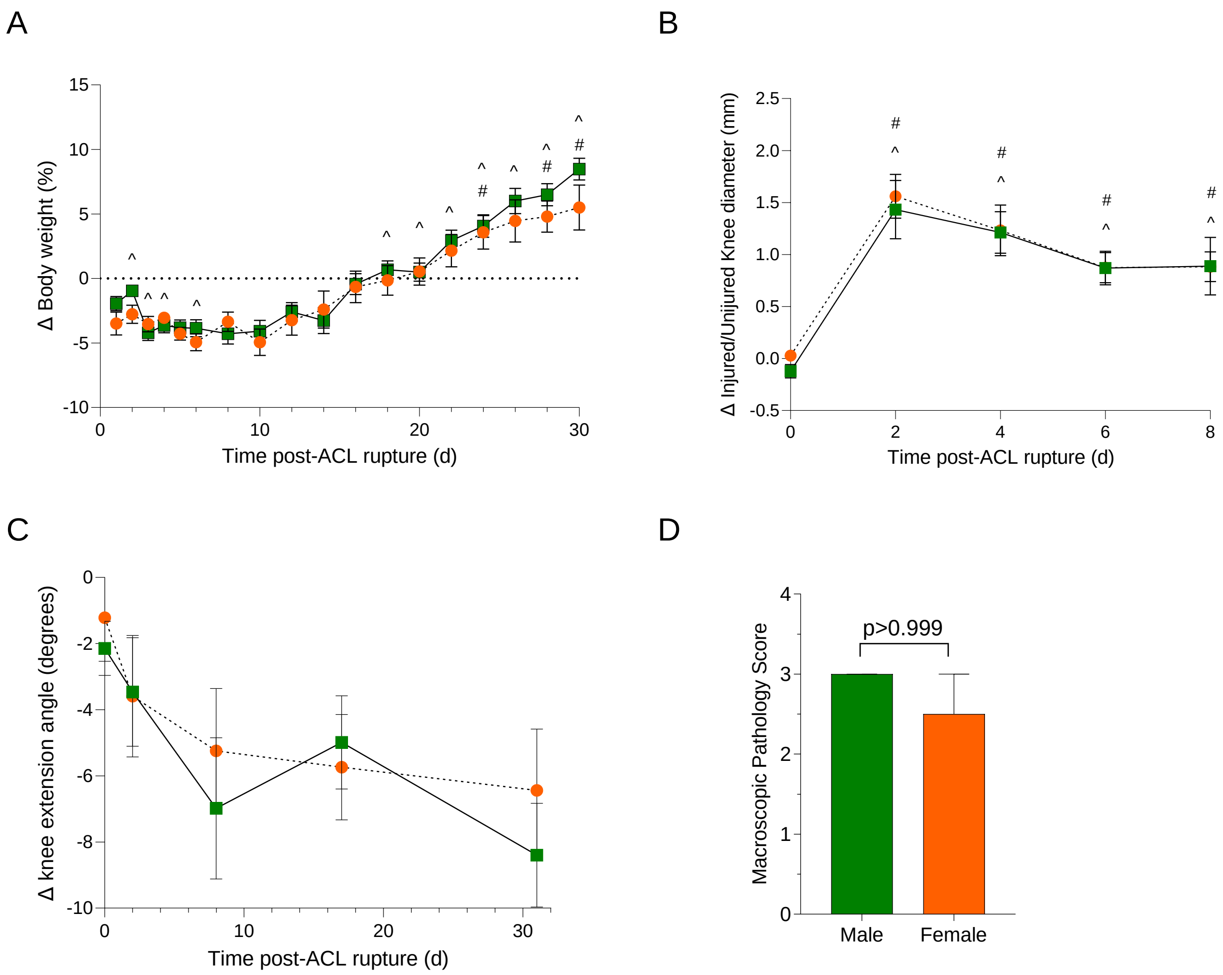

Supplement: Supplementary file 5 [file Data_Sheet_5.pdf]
