# Supplementary material for: Female rats have a different healing phenotype than males after anterior cruciate ligament rupture with no intervention
Source: Front Med (Lausanne). 2022 Nov 14;9:976980. doi: 10.3389/fmed.2022.976980 (PMC9701729; doi:10.3389/fmed.2022.976980)

Figure S2. Relative expression of key genes involved in ECM remodeling in A-D) ACL remnant and E-I) IFP of male and female ACL-ruptured and contralateral control knees at 31-days. Elastin (Eln), aggrecan (Acan), connective tissue growth factor (Ccn2), matrix metalloproteinase 9 (Mmp9), a disintegrin and metalloproteinase with thrombospondin motifs 4 (Adamts4), and tissue inhibitor matrix metalloproteinase 1 (Timp1). Data show median ± IQR. Mann-Whitney U test, *$p < 0.05$.

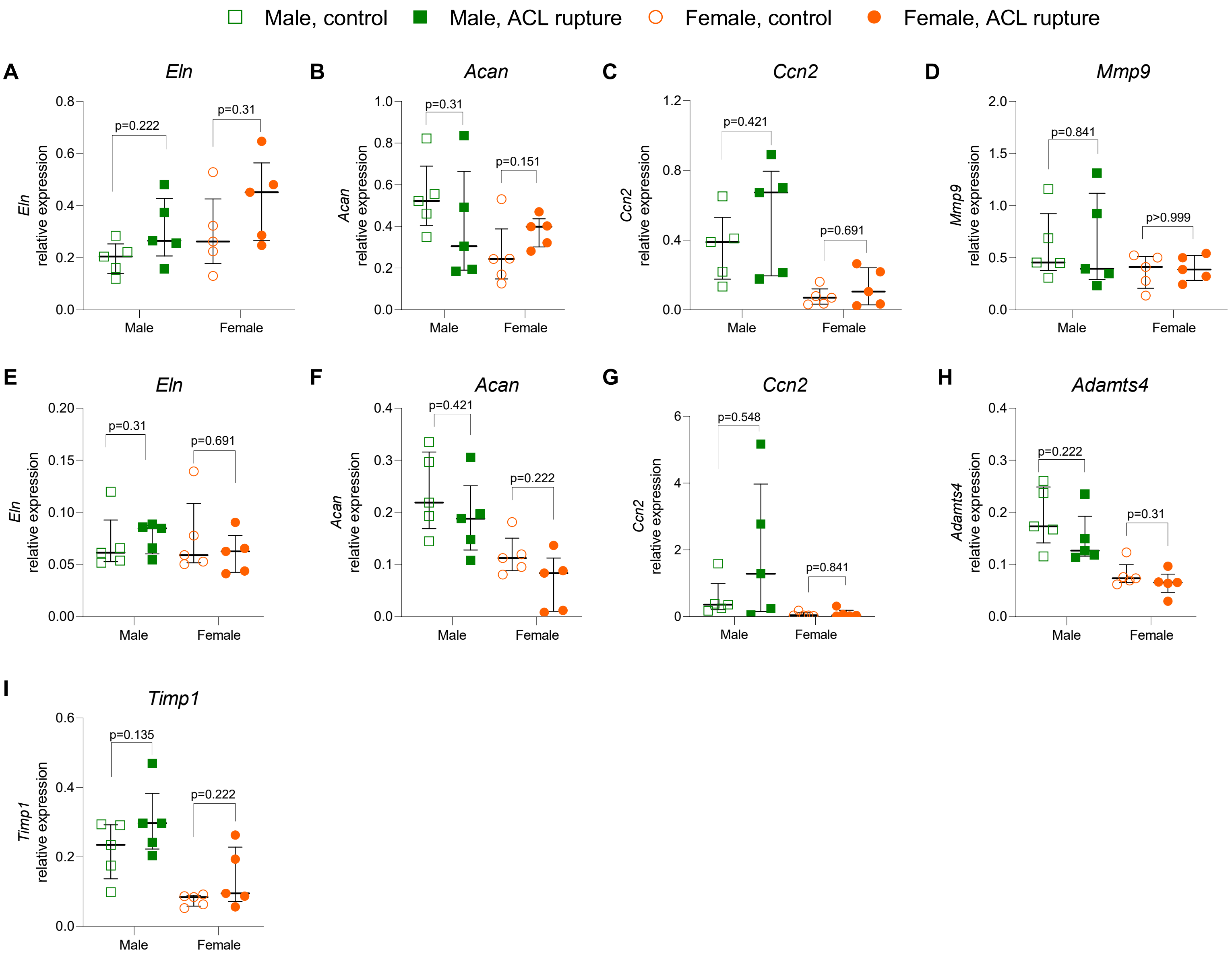

Supplement: Supplementary file 6 [file Data_Sheet_6.pdf]
