# Supplementary material for: Female rats have a different healing phenotype than males after anterior cruciate ligament rupture with no intervention
Source: Front Med (Lausanne). 2022 Nov 14;9:976980. doi: 10.3389/fmed.2022.976980 (PMC9701729; doi:10.3389/fmed.2022.976980)

Figure S4. Relative expression of key genes involved in inflammation and ECM remodeling in cartilage of the medial meniscus (MM) of male and female ACL-ruptured and contralateral control knees at 31-days. A) nuclear factor kappa B (Nfkb), B) arginase 1 (Arg1), C) elastin (Eln), D) aggrecan (Acan), E) connective tissue growth factor (Ccn2), F) matrix metalloproteinase 9 (Mmp9), G) a disintegrin and metalloproteinase with thrombospondin motifs 4 (Adamts4), H) tissue inhibitor of matrix metalloproteinase 1(Timp 1), I) transforming growth factor beta (Tgfb1), J) alpha smooth muscle actin (Acta2), and K) peroxisome proliferator activated receptor gamma (Pparg). Data show median ± IQR. Mann-Whitney U test, *$p < 0.05$.

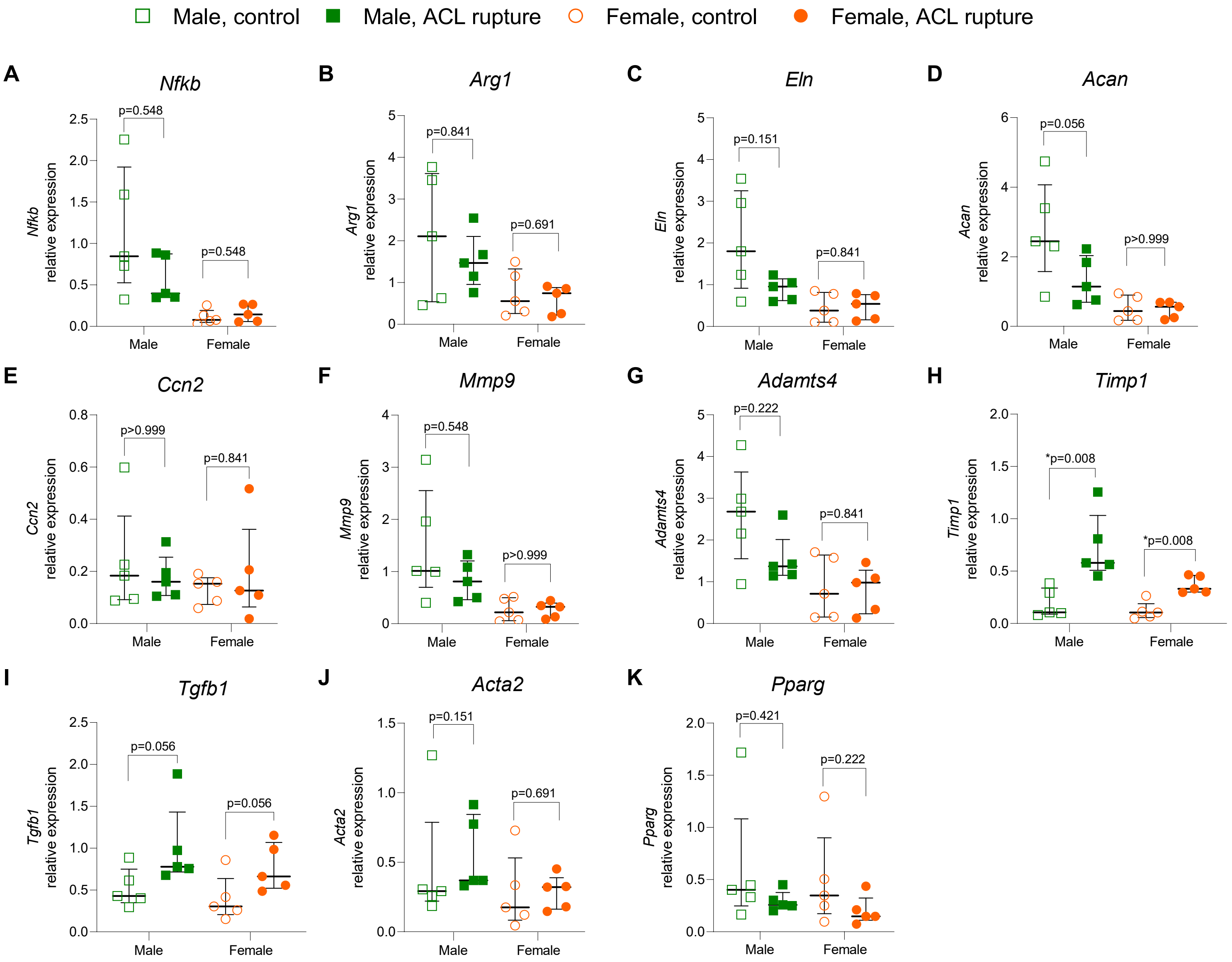

Supplement: Supplementary file 8 [file Data_Sheet_8.pdf]
